# Arrhythmogenic Right Ventricular Dysplasia and Brugada Syndrome Overlap

**DOI:** 10.7759/cureus.14482

**Published:** 2021-04-14

**Authors:** Hussein Rabah, Ali Rabah

**Affiliations:** 1 Internal Medicine, Northwell Health, New York, USA; 2 Division of Electrophysiology, Beirut Cardiac Institute, Beirut, LBN

**Keywords:** arvd, brugada ecg pattern, overlap syndrome, ecg

## Abstract

Arrhythmogenic right ventricular dysplasia (ARVD) and Brugada syndrome( BS) are associated with an increased risk of sudden cardiac death. Although they are described as two different entities, research suggests that they are not entirely separate. This paper presents a 55 years old male who presented for syncope. Interestingly, his electrocardiogram met the diagnostic criteria for both ARVD and BS. Subsequently, an implantable cardioverter-defibrillator (ICD) was implanted before discharge due to his high risk of sudden cardiac death. This case revealed that ARVD and BS clinical features can coexist in a single patient, and therefore supports the existence of a common pathophysiological basis of both diseases.

## Introduction

Arrhythmogenic right ventricular dysplasia (ARVD) is an inherited cardiomyopathy first described by Fontaine et al. in 1977 [1]. It is characterized by replacing the right ventricular myocardium with fatty tissue associated with ventricular dysfunction and arrhythmias [2,3]. In contrast, Brugada syndrome (BS) is a functional heart disease characterized by a typical EKG pattern of right bundle branch block and ST-segment elevations in the right precordial leads [4]. As opposed to patients with ARVD, patients with BS typically have no structural heart disease detectable by imaging or angiography [5,6]. However, it is generally known that there are clinical and pathophysiological similarities between ARVD and BS, but little is known about such an overlap. Therefore, in order to further understand such an overlap, we report here, a case of an overlapping state of ARVD and BS with unique electrocardiographic features. 

## Case presentation

**Figure 1:**
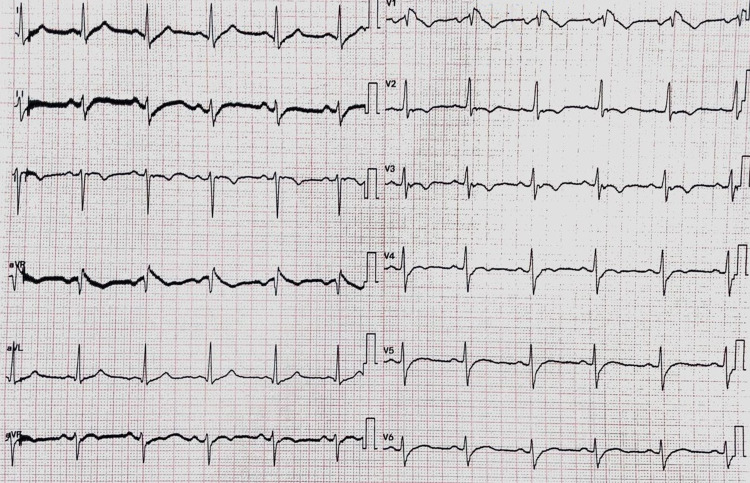
The patient's 12 lead electrocardiogram (EKG).

**Figure 2:**
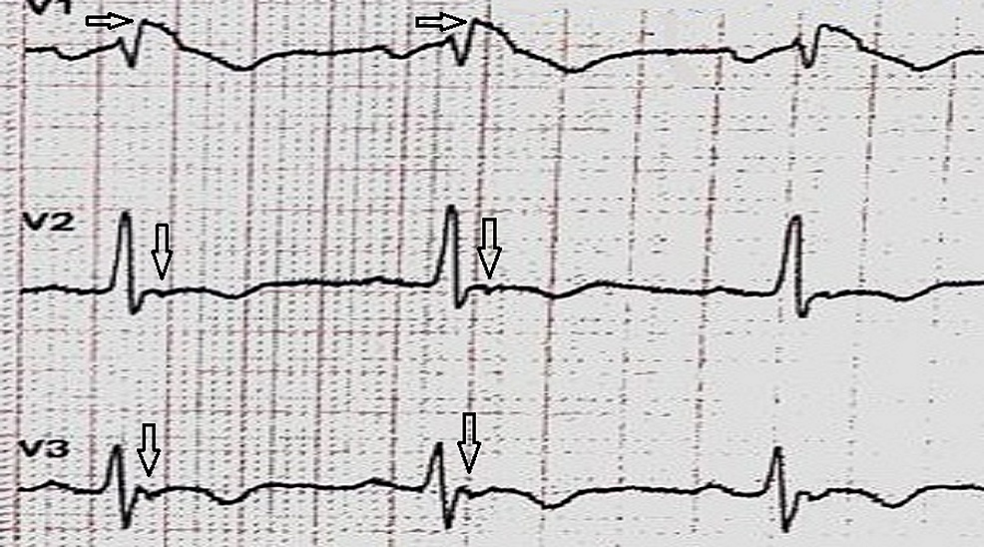
The patient's right precordial leads. Note the ST-segment elevation in v1 consistent with Brugada type 1 EKG, the "epsilon wave" shown by the black arrows, and T wave inversion evident in the leads reflecting repolarization abnormalities.

A 55-year-old man presented for syncope. Subsequently, he was admitted to the hospital for evaluation and workup. His past medical and surgical histories were unremarkable. He denied any family history of sudden cardiac death. Electrocardiogram (EKG) (Figure 1) showed right bundle branch block and ST-elevation in lead v1 consistent with Brugada type 1 EKG (Figure 2), besides an epsilon wave and T waves inversions in the right precordial leads v1, v2, v3 consistent with ARVD (Figure 2). Echocardiography showed a basal right ventricular free wall aneurysm and dyskinesis. As a result, the patient was diagnosed with ARVD after meeting the two major EKG parameters of the 2010 revised task force criteria (TFC): epsilon waves in the right precordial leads implying a depolarization abnormalities, and the inverted T waves in v1, v2, and v3 signifying a repolarization anomalies. His imaging findings met a single minor criterion, not enough for ARVD diagnosis. Interestingly, he also fulfilled the diagnostic criteria of BS as he had syncope in the presence of ST elevations in v1 consistent with BS EKG type 1. The patient was discharged from the hospital after the insertion of an implantable cardioverter-defibrillator (ICD) due to his high risk of sudden cardiac death.

## Discussion

The patient described in this report had a unique surface electrocardiogram (EKG) (Figure 1 ). He met the EKG criteria for both BS and ARVD. He exhibited ST-segment elevations in V1 consistent with BS type 1 (Figure 2) [5]. Also, epsilon waves and T waves inversions were evident on the right precordial leads consistent with the diagnostic criteria of ARVD (Figure 2) [6]. Differentiating both diseases is based on the presence or absence of clinical right ventricle structural abnormalities.

Regarding this patient's echocardiography, it revealed a right ventricular basal free wall aneurysm and dyskinesis. According to the 2010 TFC, those findings met only a minor criterion for ARVD diagnosis. Therefore, he was diagnosed with ARVD by fulfilling two major EKG criteria: apparent epsilon waves and T wave inversions in v1, v2, and v3. However, the ST-elevation in lead v1 and syncope are manifestations of an overlapping BS.

Although those diseases are described as two different entities, research supports that they are not entirely separate. Cox et al. concluded that desmosomal mutations were present in most patients with ARVD [7]. Interestingly, Cerrone et al. described that disruption of desmosomal integrity could alter sodium current and coincide with BS phenotype [8]. Pascual et al. experiment proposed that both ARVD and BS are diseases of connexons [9]. This supports that both diseases share a common molecular background and may explain the unique characteristics of the EKG described in this report. The molecular changes affected the myocardium's electrophysiological characteristics and were evident on the surface EKG as a double manifestation of the two diseases. 

On the other hand, Martini et al. described six patients who experienced ventricular fibrillation (VF) and clinical evidence of right ventricle (RV) structural abnormalities; Brugada-like ECG patterns were recognized in three patients [10]. In 1996, Corrado et al. reported a family with Brugada-like ST-segment elevation, RV cardiomyopathic changes on echocardiography, and diagnostic histopathological features of ARVC [11]. Again, this opens the possibility that both diseases represent two poles of a shared spectrum, ultimately leading to an increased risk of sudden death. However, none of the described patients had simultaneous EKG manifestations of ARVD and BS similar to our patient.

Finally, clinical, EKG, and echocardiographic features discussed in this paper might point to an overlap syndrome between ARVD and BS. Nevertheless, such an overlap syndrome’s characteristic features are still unclear, and a treatment approach to such patients is unknown. Therefore, patients with such phenotype should be identified. Due to the features of the high risk of sudden cardiac death in the patient described, including spontaneous BS type 1 EKG pattern and an episode of syncope, an ICD was implanted before discharge. 

## Conclusions

Patients with an overlapping ARVD/Brugada state should be identified to further clarify the pathological bases of such an entity. Besides, as the clinical features are not well established, careful inspection and analysis of EKGs in patients with ARVD or BS should be done looking for any overlapping features.
